# Unsuspected serotonin toxicity in the ICU

**DOI:** 10.1186/s13613-016-0186-9

**Published:** 2016-09-02

**Authors:** Catharina E. van Ewijk, Gabriel E. Jacobs, Armand R. J. Girbes

**Affiliations:** 1 Department of Intensive Care 7D16, VU University Medical Center, PO Box 7057, 1007 MB Amsterdam, The Netherlands; 2 Department of Psychiatry, VU University Medical Center, Amsterdam, The Netherlands; 3 Centre for Human Drug Research, Leiden, The Netherlands

**Keywords:** Serotonin toxicity, Serotonin syndrome, Intensive care, Delirium, Medication, Opioids

## Abstract

**Background:**

Delirium is a frequently occurring syndrome in patients admitted to the intensive care unit (ICU) or medium care unit (MCU), yet the pathophysiology remains poorly understood. An excess of central serotonin can lead to an altered mental status, associated with autonomic hyperactivity, and neuromuscular excitation. Drugs with serotonergic properties are frequently and for prolonged periods administered to ICU/MCU patients. Therefore, central serotonergic toxicity may constitute a predisposing, contributing or precipitating factor in the emergence of delirium. The purpose of the present study is to determine the number of patients admitted to the ICU or MCU who are diagnosed with delirium and who show characteristics of serotonin toxicity in association with the administration of serotonergic drugs.

**Methods:**

During a 10-week prospective observational cohort study in the ICU and MCU, patients aged 18 or older, diagnosed with delirium in the ICU or MCU, were included. Patients were considered as delirious in case of a positive CAM-ICU and/or at the start of haloperidol prescription on suspicion of delirium. Once included, patients were screened for recent administered serotonergic drugs and screened for physical signs associated with serotonin toxicity by a standardized physical examination by a specifically trained physician.

**Results:**

A total of 61 patients diagnosed with delirium were enrolled. In 44 out of 61 patients (72 %), the use of drugs potentially contributing to serotonergic toxicity was recorded. Out of 44 patients, seven (16 %) patients showed physical signs of serotonin toxicity and in addition met the Hunter serotonin toxicity criteria, suggesting the presence of serotonergic toxicity. None of these patients were recognized as such by the treating physicians.

**Conclusions:**

A significant proportion of delirious patients in the ICU might in fact be classified as suffering from central serotonin toxicity. The awareness of potential serotonin toxicity is low among physicians.

## Background

‘Altered mental status’ is a frequently occurring symptom in patients admitted to the intensive care unit (ICU) or medium care unit (MCU). Most of these altered mental states are clinically diagnosed as delirium. Delirium, mostly symptomatically treated with haloperidol, is considered to result from transient organic cerebral dysfunction and presents as an acute neuropsychiatric syndrome characterized by a disturbance in attention and cognition that tends to fluctuate during the course of the day. The reported incidence of delirium in the ICU varies between 16 and 89 % [1]. Although it is clear that delirium has a multifactorial nature, the pathophysiology remains poorly understood. Regardless of the cause, delirium is a severe syndrome and associated with many negative outcomes such as longer ICU and hospital admission, increased mortality, decreased long-term cognitive function and in addition in elderly patients with dementia increased rates of cognitive decline and admission to institutions [2–4]. In this respect, the length of duration of delirium seems to be an important prognostic factor: A longer duration of delirium is associated with worse global cognition and executive function [5].

Several mechanisms are thought to contribute to delirium, such as inflammation, metabolic derangements, electrolyte disorders, stress response and genetic factors, medication, along with imbalance of neurotransmitters [3, 6]. Regarding neurotransmitters, most research has been done on the importance of reduced acetylcholine and excess dopamine in the pathogenesis of delirium. However, an excess of the serotonergic neurotransmission, as seen in the context of an intentional overdose with serotonergic drugs, can also produce an altered mental state such as delirium, suggesting a role of serotonin (5-hydroxytryptamine or 5-HT) in the pathophysiology of delirium [6].

Nonetheless, little to no research has been conducted on this subject.

Drugs with serotonergic properties have the ability to increase the level of serotonin or to act as direct agonists of postsynaptic serotonin receptors in the central nervous system (CNS) and therefore the ability to create serotonergic toxicity. Due to the vulnerable state of critically ill patients and altered pharmacokinetics with changes in distribution volumes, protein binding and changes in blood brain barrier, serotonergic drugs might have a greater effect on serotonergic neurotransmission than we are aware of, consequently leading to toxicity more easily. To date, several drugs are known to have serotonergic properties. Most commonly known serotonergic drugs are antidepressants, such as serotonin reuptake inhibitors (SRIs) and tricyclic antidepressants (TCAs), though these drugs are not very often prescribed to patients in the ICU or MCU. An excess of CNS 5-HT, known as serotonin syndrome or serotonin/serotonergic toxicity, can lead to an altered mental status (e.g., restlessness, agitation, delirium), autonomic hyperactivity and instability (e.g., tachycardia, fluctuating blood pressure, diaphoresis, diarrhea) and neuromuscular excitation (e.g., hyperreflexia, myoclonus, rigidity). Serotonergic toxicity (ST) in itself is generally associated with a favorable prognosis. Most cases report an overdose of one or more serotonergic drugs, but toxicity can also result from therapeutic drug use or inadvertent interactions between drugs [7].

Treatment mainly requires discontinuation of serotonergic drugs and supportive care. However, most physicians are not aware of drugs that do seem to have serotonergic properties but are not evidently marked so. Yet some of them, to the contrary, are being frequently used in the ICU and MCU. Opioid analgesics (e.g., fentanyl and tramadol) and anti-emetics (e.g., ondansetron-like drugs and metoclopramide) are examples of such drugs. In addition, methylene blue and the antibiotic linezolid, which are used occasionally, are associated with inducing serotonin toxicity [7, 8]. Despite the fact that some of these drugs with serotonergic properties are frequently administered for a longer period of time to patients in the ICU and MCU, most physicians currently do not consider serotonergic toxicity as a possible cause of delirium. Since delirium in ICU and MCU patients is most often very persistent and therapy resistant, gaining understanding of the pathophysiology of delirium is evidently of utmost importance for creating better patient outcomes. We therefore set up an observational prospective cohort study to determine whether delirious patients admitted to the ICU and MCU show characteristics that possibly match serotonergic toxicity, in order to gain more clarity on whether or not serotonergic toxicity should be considered as one of the contributing factors in delirious patients.

## Methods

This study was approved by the local medical ethical committee of VU Medical Centre of Amsterdam, which waived the need for informed consent since no interventions were carried out and only non-traceable data were collected.

### Study design and study population

We performed a prospective observational cohort study during a period of 10 weeks at the ICU and MCU for adults of VU University medical center in Amsterdam. Patients aged 18 years or older, with a positive Confusion Assessment Measurement for the ICU (CAM-ICU), were included in the study. CAM-ICU is the most widely used validated instrument to detect delirium in critical ill patients, both mechanically and not mechanically ventilated patients [9, 10]. Furthermore, patients who received haloperidol on prescription of physicians on the indication of presumed delirium were considered to be delirious and were subsequently included, even in case of a negative CAM-ICU. Haloperidol is only prescribed by physicians in our department in case of suspected delirium. Patients could only be included once to minimize selection bias. Patients who had a prolonged admission and who were diagnosed with delirium previously before the start of the study, as well as patients diagnosed with delirium before admission to the ICU or MCU, were excluded.

Once included, demographic characteristics, such as age, gender, and severity of illness expressed in Acute Physiology and Chronic Health Evaluation (APACHE) II and IV score, were collected as routinely present in the patient data management system (Metavision^®^).

To determine the presence of serotonergic drugs, all medical charts were reviewed. We checked specifically for all drugs, which were associated with serotonin toxicity according to Boyer et al. [7] given at the day of diagnosing delirium as well as the previous week. Of these drugs, fentanyl is the standard first choice in our department for analgesia. Additionally, we checked whether drugs that would inhibit cytochrome P450 3A4 iso-enzyme (CYP 3A4) and 2D6 iso-enzyme (CYP 2D6) were administered, since fentanyl and other serotonergic and dopaminergic drugs are extensively metabolized by these iso-enzymes. In addition, we registered other co-medication such as sedatives that would alter mental status.

To determine whether included patients showed physical signs associated with serotonin toxicity, a separate standardized physical examination focusing on serotonin toxicity was performed by a specifically trained physician. We included those physical signs that were associated with serotonin toxicity according to Sternbach and Dunkley et al. [11, 12] along with vital signs such as heart rate and blood pressure. We defined tachycardia as a heart rate >90 beats per minute, hypertension as blood pressure >140/90 mmHg and fever as a central body temperature >38 °C. Furthermore, we reported whether there was fluctuation in blood pressure (>35 mmHg systolic) or heart rate (>40 beats/min). We relied on the Hunter serotonin toxicity criteria for the definition of serotonin toxicity: Meeting the Hunter criteria meant that there was suspicion of serotonin toxicity. Physicians were not informed of our findings, since it would intervene with our follow-up. Patients were followed up 1 week after the diagnosis of delirium was made to determine the whereabouts of the patients, the current mental status according to discharge letters or standard delirium scoring tools and whether haloperidol was still being administered. Furthermore, we checked whether serotonergic drugs were administered the week following diagnosing delirium.

We applied descriptive statistics to present the data.

## Results

**Fig. 1:**
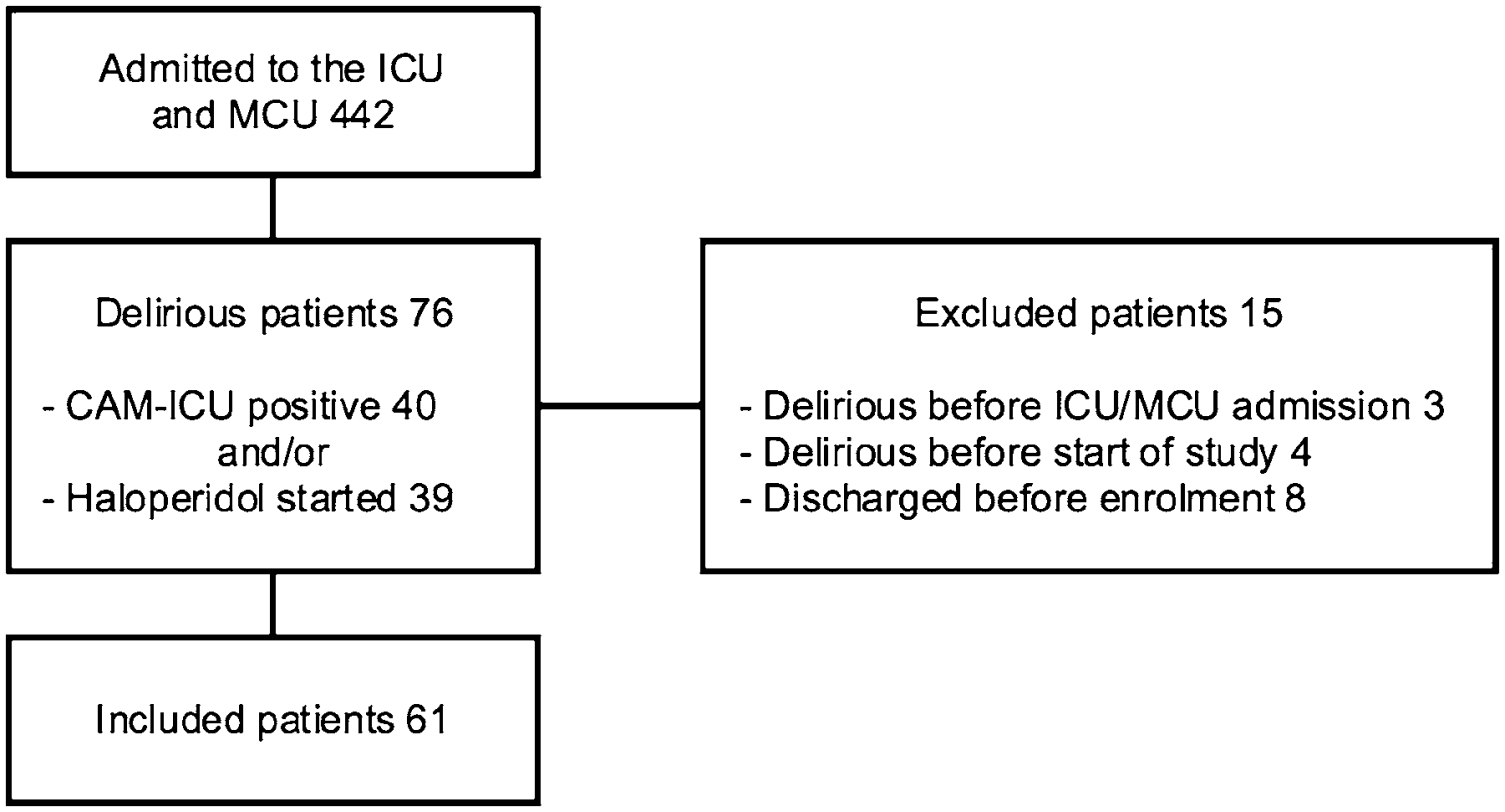
Flowchart patient inclusion

During the 10-week study period, a total of 442 patients were admitted to the ICU and MCU, of whom 76 were diagnosed with delirium, either by a positive CAM-ICU, by start of haloperidol on suspicion of delirium in case of a negative CAM-ICU or by both. A total of 15 patients were excluded, leaving 61 (Fig. 1). Once included, physical examination was performed within 48 h, with a mean of 21 h (SD 15,5 h). The characteristics of the total study population are depicted in Table 1. The cohort had a mean age of 65 and a high severity of illness. On average, the diagnosis delirium was made at day 5 (standard deviation, SD 4). Of all included delirious patients (*n* = 61), six had a history of alcohol abuse, three were admitted with acute alcohol intoxication and two had taken amphetamines before admission to the ICU. In 36 out of 61 (59 %) patients, the Richmond Agitation and Sedation Scale (RASS) was above 0 at the point of diagnosing delirium, which meant they were agitated.

**Table 1:** Characteristics of study population

Variable	*N* = 61
Age in years [mean (SD)]	65 (16)
Male [*n* (%)]	43 (71 %)
ICU admission [*n* (%)]	51 (84 %)
MCU admission [*n* (%)]	10 (16 %)
Day of admission delirium was diagnosed [mean (SD)]	5 (4)
Admission category	
Cardiac arrest [*n* (%)]	8 (13 %)
Neurology/neurosurgery [*n* (%)]	10 (16 %)
Multi-trauma [*n* (%)]	3 (5 %)
Confirmed infection [*n* (%)]	16 (26 %)
Acute kidney failure [*n* (%)]	17 (28 %)
APACHE II score [mean (SD)]	22,6 (6,8)
APACHE IV score [mean (SD)]	39 (26,2)
SOFA score [mean (SD)]	6,7 (3,2)
Comorbidity	
Psychiatric disorder [*n* (%)]	4 (7 %)
Dementia [*n* (%)]	2 (3 %)
Mechanical ventilated [*n* (%)^a^]	25 (41 %)
Richmond Agitation and Sedation Scale^b^	
Agitation (RASS > 0) [*n* (%)]	36 (59 %)
Lightly sedated (RASS ≤ 0 ≥ −2) [*n* (%)]	23 (38 %)
Moderate–heavily sedated (RASS < −2) [n (%)]	2 (3 %)
Sedation^b^ [*n* (%)]	40 (66 %)
Midazolam [*n* (%)]	13 (21 %)
Propofol [*n* (%)]	13 (21 %)
Clonidine [*n* (%)]	18 (30 %)
Benzodiazepines per os [*n* (%)]	27 (44 %)
Chronic alcohol (ab)use [*n* (%)]^c^	6 (10 %)
Acute alcohol intoxication [*n* (%)]	3 (5 %)
Chronic benzodiazepine use [*n* (%)]	8 (13 %)
Recreational drug intoxication [*n* (%)]^d^	2 (3 %)
Morphine-like analgesics [*n* (%)]	20 (33 %)
Serotonergic medication [*n* (%)]	44 (72 %)

*ICU* intensive care unit, *MCU* medium care unit, *APACHE* Acute Physiology and Chronic Health Evaluation, *SOFA* Sequential Organ Failure Assessment, *CAM-ICU* Confusion Assessment Measurement for the ICU, *SD* standard deviation, *RASS* Richmond Agitation Sedation Scale >0 = agitated

^a^At point of physical examination

^b^At enrollment

^c^More than three units/day for more than 3 months

^d^Amphetamines

In 44 out of 61 delirious patients (72 %), the use of drugs potentially contributing to serotonin toxicity was recorded at the start of the study, meaning serotonergic drugs were being administered either at the point of diagnosing delirium and/or the previous week. The drugs associated with serotonin toxicity used in the ICU and MCU are depicted in Table 2. Only those medications that were being administered during ICU admission are depicted. None of the other medication associated with serotonin toxicity according to Boyer et al. was administered.

**Table 2:** Drugs associated with serotonin toxicity used in the ICU/MCU

Serotonergic drugs	*n* = 44
Fentanyl (*n*)	36
Tramadol (*n*)	2
Metoclopramide (*n*)	7
Ondansetron (*n*)	7
CYP 3A4 inhibitor concomitantly (*n*)	16
CYP 2D6 inhibitor concomitantly (*n*)	24

*ICU* intensive care unit, *MCU* medium care unit, *n* number, *CYP 3A4 inhibitor* cytochrome P450 3A4 iso-enzyme inhibitor, *CYP2D6 inhibitor* cytochrome P450 2D6 iso-enzyme inhibitor

### Suspected serotonin toxicity

In seven (16 %) out of the 44 patients, who were delirious and had received serotonergic drugs, there were physical signs, i.e., neurological and autonomic symptoms, strongly suggesting serotonin toxicity. All seven had a positive CAM-ICU and two were receiving haloperidol as treatment of delirium. These seven patients were all given serotonergic drugs both at point of the diagnosis delirium and the whole previous week and consequently met the Hunter serotonin toxicity criteria. All seven patients were admitted to the ICU. Two were admitted after a cardiac arrest, one was admitted after high-energy trauma, one was admitted after cardiothoracic surgery, and three were admitted for internal medicine reasons. Three of the seven patients had an infection or were septic when admitted, and were treated as such, but all three were hemodynamic and respiratory stable at enrollment. One patient had a psychiatric disorder, yet did not take serotonergic psychotropics before admission, and had a history of alcohol abuse, i.e., binge drinking, but none of the seven patients was admitted with acute drug or alcohol intoxication. None of the patients with suspected serotonin toxicity had liver failure. One patient had acute kidney failure. In five out of seven patients, the RASS was above 0, in the range from agitated to combative, and six out seven patients received benzodiazepines, predominantly midazolam intravenously as treatment for agitation.

Table 3 shows the physical characteristics comparing the group with suspected serotonin toxicity (*n* = 7) to the group in which serotonin toxicity was not suspected (*n* = 54).

**Table 3:** (Physical) characteristics

Variable	Suspected ST (*n* = 7)	Not suspected ST (*n* = 54)
Age [mean (SD)]	62 (22)	65 (16)
Admission category		
Neurology/neurosurgery (*n*)	0	10
Multi-trauma (*n*)	1	2
Comorbidity		
Acute kidney failure (*n*)	1	16
Confirmed infection (*n*)	3	13
GCS [mean (SD)]	10 (4)	11 (4)
Neurological symptoms (*n*)	7	9
Especially lower extremities (*n*)	6	2
Inducible clonus (*n*)	7	1
Hyperreflexia (*n*)	3	5
Bilateral Babinski (*n*)	2	6
Autonomic symptoms (*n*)	7	35
Hypertension (>140/90 mmHg)	3	12
Fluctuating blood pressure (>35 mmHg systolic)	6	20
Vasopressin/inotropic	0	10
Tachycardia (>100 beats/min)	2	14
Sinus rhythm	7	43
Fluctuating heart rate (>40 beats/min)	1	5
Diaphoresis	3	4
Hyperthermia (>38 °C)	2	7

*ST* serotonin toxicity, *SD* standard deviation, *n* number, *GMV* Glasgow Coma Scale (eye, motor and verbal response)

As can be seen, all seven patients with suspected serotonin toxicity had an inducible clonus. One of them was admitted with traumatic brain injury, yet none of the other delirious patients admitted to the ICU because of neurological (trauma) reasons showed an inducible clonus. In all other six patients with suspected serotonin toxicity, neurological causes of clonus were excluded. In three out of seven patients, hyperreflexia was also present. In all patients but one (who was admitted with respiratory insufficiency because of a pneumonia and who showed besides an inducible ankle clonus also an inducible wrist clonus), these neurological symptoms (i.e., clonus and/or hyperreflexia) were more present at the lower extremities, a characteristic of serotonin toxicity. Concomitantly all seven patients had fluctuating blood pressure, without simultaneous administration of vasopressor or inotropic drugs. Furthermore, four out of seven patients showed diaphoresis and/or hyperthermia in the absence of an active sepsis or infection.

### No suspected serotonin toxicity

Out of the 54 delirious patients with no suspicion of serotonin toxicity (*n* = 54), only one patient had an inducible clonus, without having received any serotonergic medication. This patient was admitted because of reduced consciousness of unknown origin and was suspected of a still delirium. There was no agitation and the inducible clonus was equally present at both arms and legs, which is not a characteristic of serotonin toxicity.

Hyperreflexia was present in five patients of out 54. In only two of these patients, these symptoms were more present in the lower extremities. Diaphoresis and hyperthermia were only seen in four out of 54 and seven out of 54 patients, respectively, some of which were observed during infection. Furthermore, fluctuating blood pressure was less common (20 out of 54), and half of these patients were still supported by vasopressin or inotropic.

### Serotonergic medication prescribed at the ICU/MCU

Table 4 depicts the amount of serotonergic drugs used during ICU/MCU admission per group. All patients with suspected serotonin toxicity received intravenous fentanyl continuously. Four out of seven patients received two or more serotonergic drugs simultaneously; two received fentanyl combined with metoclopramide, one received fentanyl, metoclopramide and ondansetron, and one received fentanyl combined with tramadol. Out of the seven patients, four also received a CYP 3A4 inhibitor while receiving fentanyl, and in three cases both CYP3A4 and CYP2D6 inhibitors were administered concomitantly.

**Table 4:** Serotonergic drug use

	Suspected ST (*n* = 7)	No suspected ST (*n* = 54)
Number of serotonergic drugs concomitantly (*n*)		
None	0	17
One	3	32
Two or more	4	5
Fentanyl intravenously (*n*)	7	29
Number of days continuous [mean (SD)]	6,9 (4,7)	5,6 (4,3)
Cumulative dosage (mg) [mean (SD)]	18,5 (29,3)	14,2 (16,7)
Metoclopramide (*n*)	3	4
Tramadol (*n*)	1	1
Ondansetron (*n*)	1	6
CYP 3A4 inhibitor concomitantly (*n*)	4	12
CYP 2D6 inhibitor concomitantly (*n*)	3	21
Both CYP 3A4 and 2D6 inhibitors (*n*)	3	8

*ST* serotonin toxicity, *n* number, *SD* standard deviation, *CYP 3A4 inhibitor* cytochrome P450 3A4 iso-enzyme inhibitor, CYP2D6 inhibitor cytochrome P450 2D6 iso-enzyme inhibitor

In the group with no suspicion of serotonin toxicity, most patients received no (28 %) or only one (56 %) serotonergic drug. Only five patients received two or more drugs concomitantly, of whom three received fentanyl combined with ondansetron, one fentanyl with ondansetron and tramadol and one fentanyl combined with metoclopramide.

Twelve out of 29 received a CYP3A4 inhibitor while receiving fentanyl, 21 received CYP2D6 inhibitors, but only eight patients received CYP3A4 and 2D6 inhibitors concomitantly while receiving serotonergic drugs. 2D6 inhibitors were predominantly haloperidol, and in some cases, amiodarone, verapamil or diltiazem was administered.

### Follow-up

A week after initial enrollment, the patients with suspected serotonin toxicity were followed up. Out of seven patients, four were still admitted to the ICU or MCU, of whom three were still delirious while having received serotonergic drugs and one was not delirious anymore and had not received any serotonergic drugs the week after enrollment. The remaining three patients had been discharged to the ward, of whom one patient was still delirious while having received serotonergic drugs and two were not delirious despite having received serotonergic drugs.

## Discussion

This is to our best knowledge the first observational prospective cohort study to determine whether delirious critically ill patients who are given drugs with serotonergic properties show physical signs suggesting central serotonergic toxicity. Since a few serotonergic drugs are frequently and often for a longer period of time administered, we hypothesize that some of these delirious patients have serotonin toxicity without physicians suspecting this. In our relatively small sample, 16 % of the delirious patients who were given drugs with serotonergic properties demonstrated physical signs of serotonin toxicity and met the Hunter serotonin toxicity criteria compatible with serotonergic toxicity. Of all neurological symptoms, an inducible clonus, with or without hyperreflexia, and especially in the lower extremities, seems to be most important indication that serotonergic toxicity may be present, along with fluctuating blood pressure in the absence of vasopressor or inotropic medication. Even though half of the patients were still delirious after 1 week and serotonergic drugs were still administered, no physician suspected serotonin toxicity as a possible cause. This may suggest that physicians are not sufficiently aware of the serotonergic effects of some frequently used drugs in the ICU. Nor do they apparently expect serotonin toxicity to occur in patients who are not on antidepressants.

We used the CAM-ICU as an instrument for detecting delirium. Although it is the most widely used instrument in critically ill patients, it can lead to false positives in case of heavily sedated patients, i.e., RASS-2 or 3. A study of Heangi et al. [13, 14] showed that excluding patients with RASS-2 and 3 leads to a reduction in positives CAM-ICU of 22 %. In our cohort, the CAM-ICU assessment has only been performed in case of a RASS of -2 of higher, i.e., when a patient is lightly sedated, thus expecting few false positives.

Very little research has been done on serotonergic toxicity in the ICU. Pedavally et al. [15] performed a retrospective analysis of the clinical presentation of patients with serotonin toxicity in the ICU.

They included patients who were already diagnosed with serotonin toxicity before admission to the ICU, as well as patients who developed serotonin toxicity after admission. Most patients, who developed serotonergic toxicity during ICU admission, were on antidepressant therapy and developed symptoms indicative of serotonergic toxicity after administering additional serotonergic drugs in the ICU. Patients in our cohort did not receive antidepressants, but drugs with less serotonergic potency.

Fentanyl is a synthetic piperidine opioid with serotonergic properties. Systemic injection causes efflux of 5-HT in the dorsal raphe nucleus, either through direct 5-HT_1A_ postsynaptic stimulation or through indirect mu-opioid stimulation, which causes 5-HT reuptake inhibition [16–18]. The cytochrome P450 iso-enzyme family, especially CYP3A4, is responsible for the elimination of fentanyl. Concomitantly subscribing fluconazole or voriconazole, both CYP3A4 inhibitors, has shown to decrease the elimination of fentanyl significantly [19]. In addition, human cytochrome P450 genes are highly polymorphic, leading to inter-individual differences in drug metabolism [20].

Metoclopramide, though it is primarily a dopamine antagonist, also seems to have moderate–low affinity to 5-HT_2A_, 5-HT_3_ and 5-HT_4_ receptors [21]. Combined with fentanyl, it might enhance the risk of inducing serotonergic toxicity.

Although tramadol is not an analgesic frequently prescribed in the ICU, a recent case series by Ibister et al. [22] suggested that tramadol does not seem to provoke serotonin toxicity clinically and a study by Barann et al. [23] showed a concentration-dependent decrease in 5-HT reuptake, leading to an increase in serotonin.

Since this study contained a small cohort, no definite conclusions can be drawn on differences in drug use between the two groups. It is of note that the large majority of all our patients receive fentanyl at some stage. All patients with suspicion of serotonin toxicity had received fentanyl, started after admission to the ICU, and half of the patients had received two or more serotonergic drugs combined, mostly with metoclopramide. Yet no differences in the amount of days or total received dosage of fentanyl between the groups were present. However, four out of seven patients received a CYP3A4 inhibitor while receiving fentanyl, leading possibly to higher dosages. In addition, three out of four received besides a CYP3A4 inhibitor also a CYP2D6 inhibitor. Metoclopramide depends on CYP2D6 for inactivation. Combining CYP2D6 inhibitors (like haloperidol) with metoclopramide could therefore potentially be an extra risk factor for developing serotonergic toxicity, because of its moderate–low affinity to some postsynaptic 5HT receptors, when a patient has already been given other serotonergic drugs.

Some patients showed no signs of serotonin toxicity, despite having received multiple serotonergic agents. The most frequent combination of drugs in this group was fentanyl combined with ondansetron. Ondansetron is a 5-HT3 receptor antagonist. Turkel et al. [24] hypothesize that by blocking the 5-HT3 subtype receptor and simultaneously increasing the level of serotonin through serotonergic drugs, the excessive serotonin selectively agonizes the other serotonin subtypes, such as 5-HT1A and 5-HT2A. However, for a relevant toxicity, 5-HT levels need to be increased 40-fold over baseline [25]. Ondansetron-like drugs might, thus, not play such a big role in provoking serotonergic toxicity. Another possible explanation, for why some patients develop serotonergic toxicity and others do not, might be a combination of factors, such as unmeasured differences in severity of illness, different intra-individual susceptibility to develop serotonergic toxicity in terms of central serotonergic neurotransmission, blood–brain barrier disturbances or cytochrome P450 polymorphisms.

Serotonin toxicity is not an easy diagnosis to make. Little research has been conducted, and most is known through case reports. No gold standard diagnostic test exists, and serotonergic toxicity is essentially a phenomenological diagnosis. Dunkley et al. [12] defined the Hunter serotonergic toxicity criteria, which compared to a diagnosis made by clinical toxicologist have a sensitivity and specificity of 84 and 97 %, respectively. Although the Hunter criteria have not been developed for ICU patients and their specificity in the ICU has not been tested, we relied on the Hunter serotonin toxicity criteria.

We acknowledge that ICU patients are a difficult population to study. Many exhibit autonomic symptoms due to the critically ill condition they are in, and especially critically ill patients might have various reasons for being delirious. We have to emphasize, however, that our patients were diagnosed with delirium at the moment when they were stabilized in terms of hemodynamics and respiratory failure and sedation was minimal. Yet all seven patients showed autonomic instability and hyperactivity without active infection, support of inotropic or vasopressors, or drug/alcohol intoxication. Furthermore, the patients showed neurological symptoms that otherwise rarely occur in patients. Relying on the Hunter criteria makes misclassification unlikely.

Clinically serotonin toxicity might be difficult to distinguish from malignant neuroleptic syndrome (MNS). Excessive dopaminergic blockage can lead to MNS, characterized by fever, muscular rigidity with elevated serum CK, altered mental status and autonomic dysfunction. It is a rare idiosyncratic drug reaction to (therapeutic) doses of neuroleptic agents, and although it could in theory develop anytime, symptoms usually develop within the first 2 weeks after starting a neuroleptic agent. Serotonin toxicity, to the contrary, is a spectrum of symptoms and a toxic reaction due to an excess of serotonin in the CNS, and develops quickly after administering a serotonergic drug [26].

MNS is characterized by generalized lead pipe rigidity and bradykinesie and normal reflexia, whereas serotonin toxicity is characterized by neuromuscular hyperactivity (e.g., clonus, hyperreflexia) and predominantly in the lower extremities.

It is unlikely that the patients with suspected serotonin toxicity, in our cohort, were suffering from MNS. Haloperidol was administered to only two out of seven patients, metoclopramide to only four patients and both for a relatively short period of time. Haloperidol was one of the inclusion criteria and physical examination followed within a maximum of 48 h. Furthermore, all seven patients showed neuromuscular hyperactivity, a characteristic of serotonin toxicity and no significant elevation of serum CK. Physical symptoms started after administration of serotonergic agents, and delirium was diagnosed on average on day five of admission.

Further studies might involve a bigger cohort, possibly in comparison with non-delirious patients, to determine risk factors in terms of drug use and precipitating and predisposing factors for developing serotonin toxicity. Furthermore, it would be of use to know whether the administration of a 5-HT antagonist, such as cyproheptadine, in patients with suspicion of serotonin toxicity, would benefit in terms of symptoms that subside. Furthermore, most importantly future studies should aim to investigate whether treatment of serotonin toxicity leads to earlier recovery and better patient outcome.

In summary, we have demonstrated that a significant proportion of patients in the ICU who have been diagnosed with delirium might in fact be classified as suffering from serotonin toxicity. We furthermore showed that awareness of potential serotonin toxicity is low among physicians in our tertiary university ICU. However, lack of existing studies strongly suggests that this is not unique for our ICU. We urge for further studies on the incidence and prevalence of serotonin toxicity in ICU patients and the effect of possible early therapeutic interventions.

## Abbreviations

APACHE: Acute Physiology and Chronic Health Evaluation
CAM-ICU: Confusion Assessment Measurement for the ICU
CK: creatinine kinase
CNS: central nervous system
CYP inhibitor: cytochrome P450 isoenzyme inhibitor
GCS: Glasgow Coma Scale
5-HT: 5-hydroxytryptamine
ICU: intensive care unit
MCU: medium care unit
MNS: malignant neuroleptic syndrome
RASS: Richmond Agitation and Sedation Scale
SD: standard deviation
SOFA: Sequential Organ Failure Assessment
SRI: serotonin reuptake inhibitor
ST: serotonin toxicity
TCA: tricyclic antidepressant
